# Electron-Beam-Induced Fluorination Cycle for Long-Term Preservation of Graphene under Ambient Conditions

**DOI:** 10.3390/nano12030383

**Published:** 2022-01-24

**Authors:** Tianbo Duan, Hu Li, Klaus Leifer

**Affiliations:** 1 Ångström Laboratory, Department of Materials Science and Engineering, Uppsala University, 75121 Uppsala, Sweden; Tianbo.Duan@angstrom.uu.se (T.D.); Hu.Li@angstrom.uu.se (H.L.); 2 Shandong Technology Centre of Nanodevices and Integration, School of Microelectronics, Shandong University, Jinan 250101, China

**Keywords:** graphene, electron-beam-induced deposition, fluorination, airborne contamination

## Abstract

The aging in air inevitably results in the accumulation of airborne hydrocarbon contaminations on a graphene surface, which causes considerable difficulties in the subsequent application of graphene. Herein, we report an electron-beam-activated fluorination/defluorination cycle for achieving a long-term preservation of CVD graphene. After experiencing such cycle, the accumulation of airborne hydrocarbon on the graphene surfaces is strongly reduced, and the initial chemical status of graphene can be restored, which is confirmed by employing atomic force microscopy and X-ray photoelectron microscopy. Our reported approach provides an efficient method for the cleaning and long-term preservation of graphene, and it is particularly useful for graphene microscopy characterizations.

## 1. Introduction

The emergence of graphene, an atomic thick carbon layer with honeycomb lattices, has prompted many interesting observations and promising applications [1,2]. To achieve a controlled performance of graphene in various fields, such as surface analysis, functionalization and nanoelectronic devices, a graphene sample with superior quality is highly desirable [3,4,5]. For analyzing and processing graphene on a nanoscale, the long-term endurance of graphene to the environment is of the highest importance, especially for electron beam microscopy analysis, which often requires an ultra-clean surface of graphene to achieve atomic resolution imaging [6,7,8]. Although various studies have been carried out to minimize the contamination from graphene, the surfaces cannot be totally protected from unintentional contaminations, e.g., airborne hydrocarbon [9,10]. Airborne hydrocarbon, which mainly consists of volatile organic compounds (alkanes, alkenes, aromatics, alcohols), exists almost everywhere, even in the high-end cleanroom for nanofabrication. Moreover, several studies have shown that hydrocarbon can change the wettability of a graphene surface, which affects its performance in the synthesis process. Albeit O_2_ plasma treatment is efficient to prevent the contamination of the sample by hydrocarbon, such plasma approach is highly chemically reactive and thus can damage graphene, even with a low power input [11].The typical method to eliminate airborne hydrocarbon is through vacuum annealing, but the resulting surface is not sufficiently clean for many surface analysis purposes [12]. The annealing method is significantly limited by the insertion of defects under a relatively high temperature [13]. Moreover, the cleanness of the graphene surface cannot be preserved during prolonged aging. An efficient strategy is thus needed for the long-term preservation of graphene samples. Recently, Li [14] has proposed a reversible fluorination technique induced by an electron beam in vacuum. The study has shown that the initial graphene status can be restored after a fluorination/defluorination cycle. Herein, we report a technique to eliminate the airborne hydrocarbon on graphene during aging by using the electron-beam-activated fluorination/defluorination cycle. X-ray photoelectron spectroscopy (XPS) is carried out to analyze the accumulation of hydrocarbon both on pristine graphene and fluorinated/defluorinated graphene. The surface status of graphene is further investigated by atomic force microscopy. Moreover, we have studied the structural evolution of graphene after the fluorination/defluorination cycle by Raman spectroscopy and gated measurements.

## 2. Materials and Methods

### 2.1. Materials and Device Fabrication

The chemical vapor deposition of graphene on synthesized Cu foil is transferred onto a SiO_2_/Si substrate with a SiO_2_ thickness of 300 nm using a polymer-assisted wet chemistry method. The as-transferred graphene is then cleaned by applying a solvent (isopropanol/H_2_O for 2 min at room temperature) mixture treatment to effectively remove the polymer residues on the surface. The back-gated graphene devices are fabricated through standard electron beam lithography (EBL) in a Nanobeam nB5 EBL system, oxygen plasma etching in an Advanced Vacuum Vision 320 Reactive Ion Etching (RIE) system, and metal deposition in a Lesker PVD 75 system (Kurt J. Lesker Ltd., Jefferson Hills, PA, USA).

### 2.2. Graphene Aging and Fluorination/Defluorination

Graphene samples are kept under ambient condition with a temperature of 20 ± 1 °C and a relative humidity of 45 ± 3% for aging. The electron-beam-activated fluorination of graphene is conducted in a FEI Strata DB235 FIB/SEM (Thermofisher, Hillsboro, OR, USA) at a vacuum condition of 10^−6^ mbar under an electron acceleration voltage of 5 kV. The fluorination is carried out by the irradiation of graphene with an electron beam for 300 s with a size of 100 µm × 100 µm, while simultaneously injecting the XeF_2_ via GIS system in FIB (Thermofisher, Hillsboro, OR, USA). For the defluorination process, electron beam irradiation is carried out without gas injection for 100 min.

### 2.3. Characterizations

Raman and X-ray photoelectron spectra are acquired in Renishaw inVia Raman spectrometer (Renishaw, Kingswood, UK), with a 532 nm excitation laser, and Physical Electronics Quantera II (ULVAC-PHI, Chanhassen, MN, USA) Scanning XPS Microprobe monochrome Al Kα radiation (1486.7 eV), with a 45° angle of electron emission, respectively. The electrical measurements of the graphene back-gated devices are carried out using an Agilent B1500 semiconductor parameter analyzer (Keysight, Santa Rosa, CA, USA) inside a Faraday cage with metal probes.

## 3. Results

**Figure 1:**
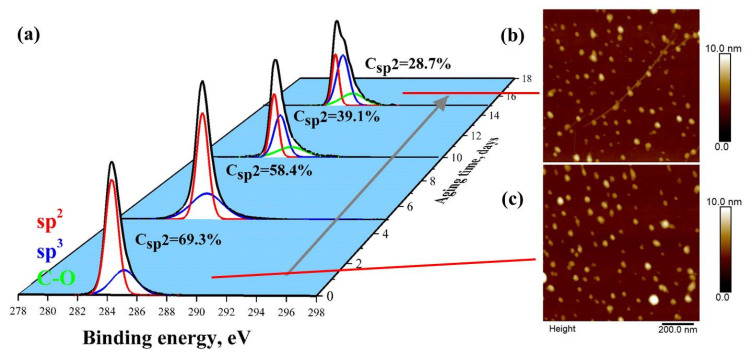
Surface characterization of graphene after aging in the air for 15 days: (**a**) C1s core level X-ray photoelectron spectra of graphene surface after aging. The peak consists of 3 main parts, which are sp^2^ carbon (red) located at 284.0 eV, sp^3^ carbon (blue) located at 284.8 eV, and C–O (green) located at 286.1 eV. Height contrast atomic force microscopy images of (**b**) graphene sample aged after 15 days and (**c**) initial graphene sample.

The graphene samples are synthesized by chemical vapor deposition (CVD) at a large scale and subsequently transferred onto a SiO_2_/Si substrate following a solvent mixture cleaning (see the details in Materials and Methods) to minimize the contamination from the polymer residues during transferring. Figure 1 shows the X-ray photoelectron spectroscopy (XPS) results of the graphene samples C1s peak after aging for 15 days at air under a cleanroom environment. The C1s peak undoubtedly consists of 2 main components: the sp^3^ C peak located at 284.8 eV and the sp^2^ C peak located at 284.0 eV. The initial larger intensity of the sp^2^ peak can be mainly attributed to the intrinsic sp^2^ hybridization of graphene, whereas the sp^3^ bonds are contributed by the defects, polymer residues, and hydrocarbon. As the aging process is underway, the concentration of sp^2^ drops from 69.3% to 28.7%, while the sp^3^ bonds continuously increase, and this is mainly attributed to the accumulation of airborne contaminations on the graphene surface. Moreover, we have observed a considerable C–O peak located at 286.1 eV after 10 days of aging which can also be ascribed to the oxygen-containing contaminations coming from the air. Figure 1b,c shows the atomic force microscopy images of the graphene aged after 15 days and the initial pristine graphene, respectively. It can been seen that there is barely no topographical change on the graphene surface after aging, indicating that the evolution of the C1s signal can be explained as the accumulation of airborne contaminations, which is difficult to characterize with a normal height image.

**Figure 2:**
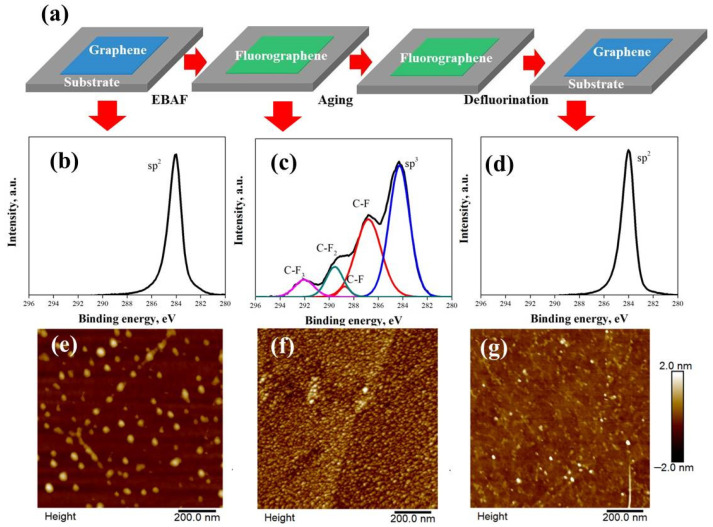
(**a**) Illustration figure of the fluorination/defluorination cycle on graphene. The C1s core level spectra at different steps are shown in (**b**–**d**), corresponding to the initial graphene, fluorinated graphene, and defluorinated graphene, respectively. (**e**–**g**) on the right show the height contrast AFM images, corresponding to the graphene at different fluorination/defluorination cycles.

Figure 2a illustrates the electron-beam-induced fluorination/defluorination cycle for eliminating the aging effect of the graphene surface. CVD graphene is fluorinated inside a SEM chamber by using electron-beam-activated fluorination. In this method, graphene is illuminated under the electron beam while simultaneously injecting XeF_2_ gas. After the aging experiment of the as-fluorinated sample in the air, the fluorinated graphene is again exposed to the electron beam without precursor gas to perform the defluorination process (more details can be found in Materials and Methods). The C1s XPS spectra of graphene at different steps of the fluorination/aging/defluorination cycle are shown in Figure 2b (before fluorination), Figure 2c (after fluorination), and Figure 2d (after defluorination). It is clear that before fluorination the C1s peak of initial graphene is mainly composed of an sp^2^ signal located at 284.4 eV, and after fluorination there are 4 main components in the spectrum: the sp^3^ peak (284.8 eV), C–F peak (287.4 eV and 289.2 eV), C–F_2_ peak (290.0 eV) and C–F_3_ peak (292.0 eV). The absence of sp^2^ suggests the saturated fluorination of graphene. Figure 2e shows the XPS spectrum of graphene after the defluorination process. Interestingly, we have observed that the C1s peak is composed of the sp^2^ signal, and the sp^3^ intensity is relatively low, similarly to that of the initial graphene sample. In order to confirm the reproducibility of this result, more C1s XPS spectra with corresponding AFM images of defluorinated graphene under the same process have been shown in Figure S1. The XPS results clearly illustrate the effect of the fluorination/defluorination cycle on eliminating the airborne contamination on the graphene surface resulting from the aging process. As several studies have already shown that electron beam irradiation can effectively improve the removal of polymer residues by decomposition [15,16], the mechanism of the airborne contaminants removal during electron beam irradiation is rarely reported. In the work, during the defluorination process, the C–F bonds with an ionization cross section of up to 80 eV can be dissociated with both backscattered and secondary electrons [17,18] to generate fluorine radicals. As shown in Figure S2, the F1s peak has disappeared after experiencing the fluorination/defluorination cycle, indicating that the fluorine radicals have evaporated in the high vacuum of the SEM chamber. We believe the cleaning effect can be attributed to the interactions between fluorine radicals and airborne contaminants on the surface during the electron beam irradiation.

To further investigate the evolution of the surface status of graphene during the fluorination/defluorination cycle, we have carried out an AFM analysis on the graphene surface at different processing steps, shown by Figure 2e (before fluorination), Figure 2f (after fluorination), and Figure 2g (after defluorination). It can be observed that the surface roughness of graphene has increased and the height of the impurities has decreased after fluorination. There is a large number nano-sized fluorinated clusters, which have been reported before [19,20]. Most of these clusters disappear after the defluorination process, as observed in Figure 2g, indicating the restoring of the initial status of graphene. Moreover, the number of polymer residues with a size of 30–100 nm has decreased after fluorination/defluorination, which is in agreement with the previously reported decomposition effect, and may result in a more contamination-free status of graphene.

**Figure 3:**
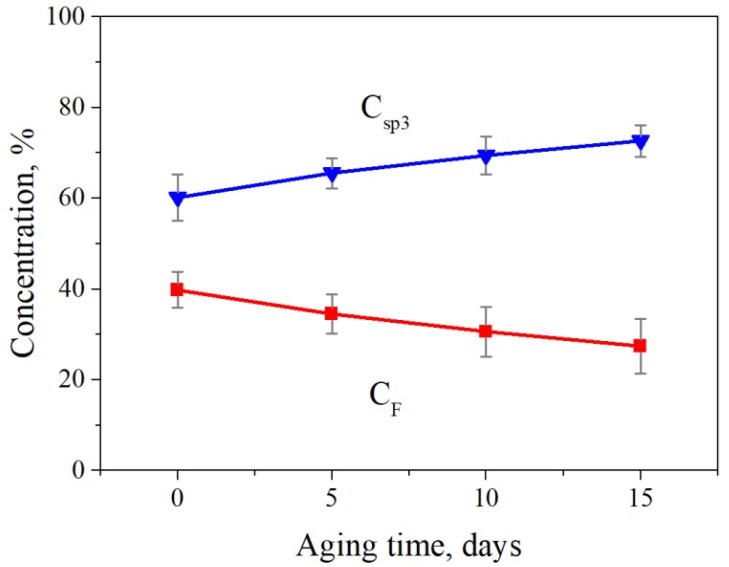
The concentrations of sp^3^ bonds C_sp3_ and C–F bonds C_F_ plotted with the aging time. It can be clearly seen that there is an increase of sp^3^ intensity after aging as a consequence of the accumulation of hydrocarbon, and the corresponding F bonds decrease accordingly.

As the accumulation of hydrocarbon will inevitably happen on the fluorinated graphene, the concentrations of sp^3^ bonds C_sp3_ and C–F bonds C_F_ with different aging time are shown in Figure 3. We observe that C_sp3_ slightly and linearly increases from 60.2 ± 5.1% to 72.6 ± 3.5%, whereas C_F_ drops accordingly with time increases. This result indicates the stability of fluorographene during aging, and it provides further proof of the hydrocarbon accumulation even on the fluorinated surface during aging.

**Figure 4:**
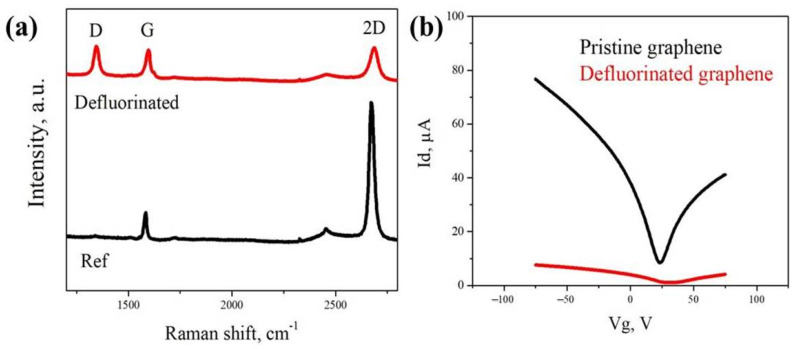
Characterizations of graphene after the fluorination/defluorination cycle: (**a**) shows the Raman spectra of initial pristine graphene and defluorinated graphene. The emerging D peak after defluorination suggests the introduction of disordering as a result of the electron beam irradiation; (**b**) shows the gated curves of pristine graphene (in black) and defluorinated graphene (in red).

In order to investigate the effect of the fluorination/defluorination cycle on graphene properties, the Raman spectroscopy and gated measurements have been carried out. Figure 4a shows the Raman spectra of the initial graphene and fluorination/defluorination treated graphene. It can be seen that after defluorination, there is an emerging D peak (located at 1350 cm^−1^) besides the intrinsic G peak (located at 1580 cm^−1^) and 2D peak (located at 2690 cm^−1^), indicating the introduction of disordering after defluorination. Moreover, the broadening of the 2D peak also suggests the structural changes in graphene. This disordering can be mainly attributed to the damage from electron beam irradiation [21,22]. Figure 4b shows the gated measurements of pristine graphene and defluorinated graphene with the source-drain voltage of 50 mV. We have observed a significant decrease of conductivity after defluorination, and the charge carrier mobility drops from 5811 cm^2^ V^−1^ S^−1^ to 233 cm^2^ V^−1^ S^−1^. The substantial drop in conductivity and mobility has further provided the evidence of the disordering after electron beam irradiation during defluorination. As the transport behavior of graphene can be mainly affected by the grain boundaries [23], the decrease of mobility in this case can be attributed to the grain boundaries’ degradation after defluorination.

In conclusion, we have reported a novel technique for achieving a long-term preservation of supported graphene by using an electron-beam-activated fluorination/defluorination cycle. In this research, the accumulation of hydrocarbon on CVD graphene due to the aging in the air has been observed and characterized by an XPS analysis, with the concentration of the sp^2^ signal dropping from 66.1% to 26.2%. We have demonstrated that by implementing the electron-beam-activated fluorination/defluorination cycle, the hydrocarbon accumulation can be eliminated, which restores the initial chemical status of graphene before fluorination. Moreover, we show that after the defluorination cycle the disordering is introduced due to the electron beam irradiation with an emerging D peak in Raman spectra and the degradation of mobility in gated measurements.

## Data Availability

Experimental data is available from the authors upon reasonable request.

## Supplementary Materials

The following supporting information can be downloaded at: https://www.mdpi.com/article/10.3390/nano12030383/s1, The XPS and AFM analyses of the graphene sample after the fluorination and defluorination cycle. Figure S1: (a) and (c) are C1s XPS spectra of defluorinated graphene experienced the same fluorination/defluorination cycle and (b) and (d) are corresponding AFM images, respectively. Figure S2: F1s XPS spectra of graphene at different fluorination/defluorination stage.
