# Supplementary material for: Electron-Beam-Induced Fluorination Cycle for Long-Term Preservation of Graphene under Ambient Conditions
Source: Nanomaterials (Basel). 2022 Jan 24;12(3):383. doi: 10.3390/nano12030383 (PMC8839107; doi:10.3390/nano12030383)
Supplement: Supplementary file 1 [file nanomaterials-12-00383-s001.zip › nanomaterials-1529863-supplementary.pdf]

nanomaterials

Supplementary Materials

Electron-Beam-Induced Fluorination Cycle for Long-Term Preservation of Graphene under Ambient Conditions

Tianbo Duan [1], Hu Li [1,2] and Klaus Leifer [1,*]

[1] Ångström Laboratory, Department of Materials Science and Engineering, Uppsala University, 75121 Uppsala, Sweden; Tianbo.Duan@angstrom.uu.se (T.D.); Hu.Li@angstrom.uu.se (H.L.)

[2] Shandong Technology Centre of Nanodevices and Integration, School of Microelectronics, Shandong University, Jinan 250101, China

* Correspondence: Klaus.Leifer@angstrom.uu.se

Electronic supplementary information

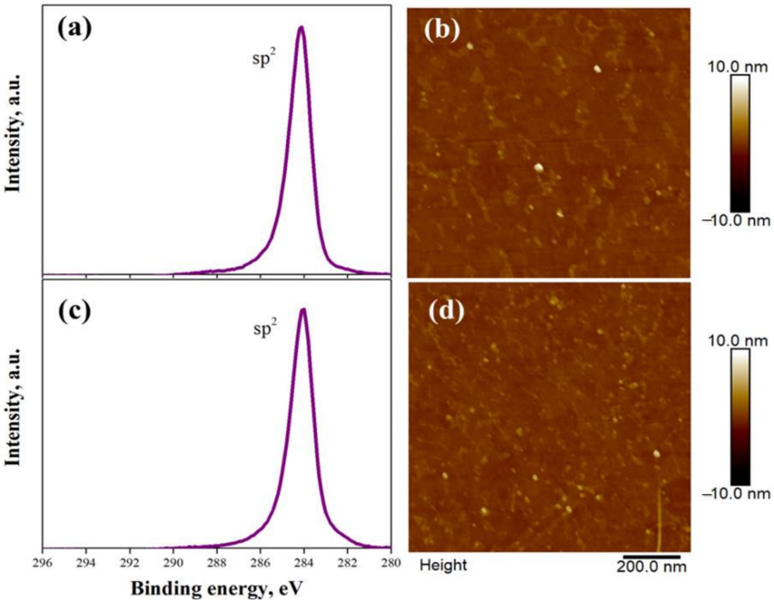

Figure S1. (**a**) and (**c**) are C1s XPS spectra of defluorinated graphene experienced the same fluorination/defluorination cycle and (**b**) and (**d**) are corresponding AFM images, respectively.

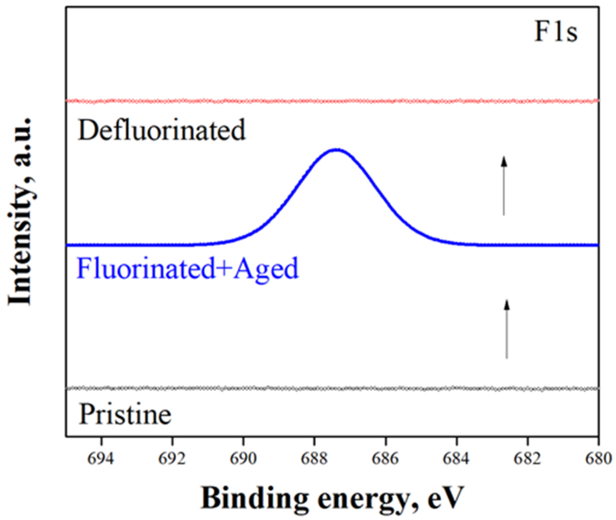

Figure S2. F1s XPS spectra of graphene sample at different fluorination/defluorination stage.
